# Identification of prognosis markers for endometrial cancer by integrated analysis of DNA methylation and RNA-Seq data

**DOI:** 10.1038/s41598-019-46195-8

**Published:** 2019-07-09

**Authors:** Xiao Huo, Hengzi Sun, Dongyan Cao, Jiaxin Yang, Peng Peng, Mei Yu, Keng Shen

**Affiliations:** Department of Obstetrics and Gynecology, Peking Union Medical College Hospital, Chinese Academy of Medical Sciences and Peking Union Medical College, Beijing, China

**Keywords:** Tumour biomarkers, Prognostic markers

## Abstract

Endometrial cancer is highly malignant and has a poor prognosis in the advanced stage, thus, prediction of its prognosis is important. DNA methylation has rapidly gained clinical attention as a biomarker for diagnostic, prognostic and predictive purposes in various cancers. In present study, differentially methylated positions and differentially expressed genes were identified according to DNA methylation and RNA-Seq data. Functional analyses and interaction network were performed to identify hub genes, and overall survival analysis of hub genes were validated. The top genes were evaluated by immunohistochemical staining of endometrial cancer tissues. The gene function was evaluated by cell growth curve after knockdown CDC20 and CCNA2 of endometrial cancer cell line. A total of 329 hypomethylated highly expressed genes and 359 hypermethylated lowly expressed genes were identified, and four hub genes were obtained according to the interaction network. Patients with low expression of CDC20 and CCNA2 showed better overall survival. The results also were demonstrated by the immunohistochemical staining. Cell growth curve also demonstrated that knockdown CDC20 and CCNA2 can suppress the cell proliferation. We have identified two aberrantly methylated genes, CDC20 and CCNA2 as novel biomarkers for precision diagnosis in EC.

## Introduction

Endometrial cancer (EC) is a gynecological cancer that is commonly diagnosed in developed countries, accounting for approximately 7% of new cancer cases and 4% of cancer-related deaths in women^1^. Most women diagnosed at an early stage have a long survival time; however, those with high-risk histopathology or at an advanced stage have a poor prognosis^2^. Early diagnosis, reasonable assessment of prognosis and timely intervention are important. Although the Federation International Of Gynecology and Obstetrics (FIGO) staging system combined with histology help us to manage the EC patients, it remains sufficient to accurately predict the prognosis due to the molecular heterogeneity of EC^3^. Therefore, there is an urgent need to identify sensitive and specific molecular markers for prognosis to achieve personalized treatment and improve clinical outcomes. Many studies have focused on specific molecular alterations in EC, such as gene mutations, DNA methylation, microsatellite instability, and copy number alterations^4–8^.

DNA methylation is a major epigenetic mechanism that inhibits the binding of transcription factors or the recruitment of inhibitory proteins, which is closely related to normal development and cellular function, including embryonic development, regulation of gene expression, X-chromosome inactivation, genomic imprinting, and genomic stability^9–13^. Recently, DNA methylation has been extensively investigated in cancer. Cancer-specific changes include hypermethylation of C-phosphate-G (CpGs) in gene promoters, hypomethylation of non-CpG island CpGs, and an overall increase in the variation in methylation^14,15^. Cancer-associated hypermethylation of CpG islands (CGIs) in gene promoters, which induces the silencing or downregulation of genes, especially tumor suppressor genes, may contribute to tumorigenesis and progression^14^. Therefore, DNA methylation has rapidly gained clinical attention as a biomarker for diagnostic, prognostic and predictive purposes in various cancers^16,17^.

During recent decades, a few specific DNA methylation signatures associated with high-risk EC and a series of altered methylation genes associated with unfavorable prognostic factors have been identified, such as TBX2, CHST11, PTEN and NID2^18–20^. Even though advanced studies permit genome-wide screening at the DNA methylation or mRNA expression level, the mechanisms of DNA methylation that are involved in the regulation of gene expression and affect prognosis in EC remain unclear. However, although methylation studies in EC are still preclinical, the understanding of how DNA methylation is associated with the prognosis of EC should continue to develop so that we can accurately predict the prognosis and improve the survival time of EC patients. The present study investigated altered DNA methylation patterns by integrating methylomes and transcriptomes of both EC and normal tissues available in the TCGA data portal with DNA-binding proteins and their binding motifs, aiming to identify specific DNA methylation genes as potential biomarkers for predicting the prognosis of EC patients.

## Results

### Identification of DEGs, DMPs, EI and ES expression in EC

According to the screening conditions, a total of 8464 DEGs in the cancerous and paracancerous samples were obtained from the 60,483 transcripts, of which 3325 were upregulated and 5319 were downregulated. A total of 78,963 DMPs were obtained from 208,022 methylation positions in the cancerous and paracancerous samples, of which 34,637 were upregulated and 44,500 were downregulated. We mapped the DMPs to gene promoters, and in cases with multiple DMPs in the same promoter region, we chose the consistently upregulated or downregulated positions as the valid DMP for the differentially methylated gene (DMG). Finally, 3180 and 9106 DMGs were obtained by upregulated and downregulated DMP mapping, respectively. The most significant top 100 DEGs and DMGs are shown in Supplementary Fig. S1.

**Figure 1:**
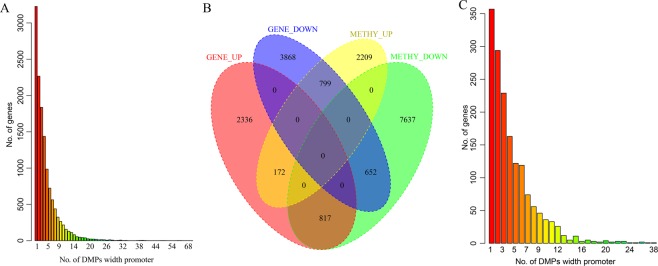
Gaussian hierarchical clustering map of the top 100 gene with the most significant difference. (**A**) The clustering map of differentially expressed genes; (**B**) differentially methylated genes.

Subsequently, the number of DMPs in the promoter region of each gene was counted, and the distribution of genes corresponding to different DMP numbers was further analyzed as shown in Fig. 1A. The promoter regions of most DMGs have only one DMP. Then, we analyzed the relationship between the DEGs and DMPs as shown in Fig. 1B, and 817 EI genes and 799 ES genes were found, of which 329 EI genes and 359 ES genes were ultimately obtained for a significantly negative correlation between DEGs and DMPs. We analyzed the distribution of genes corresponding to the different DMP numbers in the promoter regions of the 688 negatively regulated genes, as shown in Fig. 1C, in which there are significantly less EI/ES in promoter regions with only one DMP. This finding suggests that these EI/ES genes are more prone to be regulated by multiple methylation positions in the promoter regions.

### GO and pathway functional enrichment analysis

The KEGG and GO functional enrichment analyses of identified EI and ES genes were performed by the ClusterProfiler package for R. The results of the enrichment analysis revealed that EI genes were enriched in KEGG biological pathways associated with regulation of focal adhesion, proteoglycans in cancer and ECM-receptor interaction (Supplementary Fig. S2A), and ES genes are mainly enriched in pathways associated with regulation of the cell cycle, DNA replication, mismatch repair, and the p53 signaling pathway (Supplementary Fig. S2B), which are important signaling pathways. According to the GO analysis, EI genes were enriched for 188 terms, including 144 within the “biological process” category, 30 within the “cellular component” category, and 14 within the “molecular function” category. In addition, ES genes were enriched for 67 terms, including 62 within the “biological process” category, 1 within the “cellular component” category, and 4 within the “molecular function” category. Comparative analysis found that the 188 GO terms enriched among EI genes have no intersection with the 67 GO terms among ES genes, suggesting that ES and EI genes may perform different biological functions. Furthermore, we performed a crosstalk analysis on these GO terms with JACCARD ≥0.5 using the EnrichmentMap plugin for Cytoscape, and the results showed that EI genes were mainly enriched for regulation of the mitotic cycle and single-stranded DNA (Supplementary Fig. S3A) and that the ES genes were mainly enriched for regulation of proteinaceous extracellular structure and regulation of the cation transmembrane (Supplementary Fig. S3B).

### Construction and analysis of the genetic interaction subnet

The PPI network, which was downloaded from the HIPPIE database, contains 17,381 nodes, and the average number of neighboring nodes is 19.6. The genetic interaction subnet was constructed by mapping the EI and ES genes to the PPI network; a total of 570 genes were mapped into the network, of which 241 showed interactions, and the average number of neighboring nodes was 1.36. Supplementary Figure S4 shows that as the degree increased, the number of nodes was reduced.

Combined with the network topology analysis and the background PPI network, a statistical model was constructed to calculate the ES and EI enrichment of each gene in the genetic interaction subnet. The network diagram constructed by Cytoscape showed that there are fewer ES genes in the network, and most ES/EI gene enrichment was of low significance (Supplementary Fig. S5). According to the screening threshold FDR < 0.05, genes with neighboring nodes containing ≥5 EI/ES genes were defined as hub genes, and four hub genes named CCNA2, CDC20, POC1A, and CDH1 were obtained (Table 1).

**Table 1:** The hub genes in the interaction subnet.

GeneSymbol	Number of EI Neighborhood gene	Number of Neighborhood gene	Number of EI gene	Number of network gene	EI Neighborhood gene per	Fisher’s exact test pvalue	FDR
CCNA2	10	125	241	17381	0.0740740	1.89E-05	0.004548
CDC20	11	160	241	17381	0.0643274	2.73E-05	0.006543
POC1A	8	88	241	17381	0.0833333	5.64E-05	0.013491
CDH1	23	688	241	17381	0.0323488	0.000188	0.044765

### Hub gene validation

**Figure 2:**
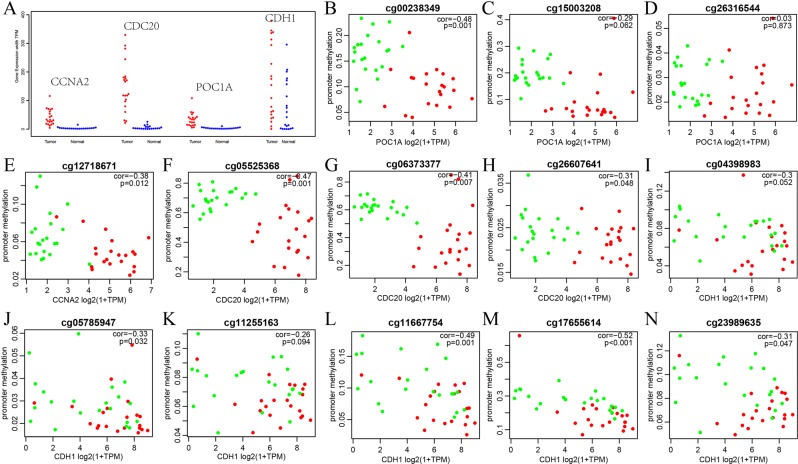
The validation of hub genes using external TCGA database. (**A**) Expression distribution of four genes in cancer and adjacent tissues; (**B**–**N**) Relationship between the expression level of the differentially promoter methylation positions and the expression level of hub genes. Red dots indicate cancer samples and green dots indicate paracancerous samples.

The four hub genes were then validated using the external TCGA database to confirm the validity of our findings. First, we analyzed the expression distribution of these four genes in cancer and adjacent tissues as shown in Fig. 2A. The expression distributions of CCNA2, CDC20 and POC1A were relatively centralized. However, CDH1 had a more dispersed distribution in different samples, which suggests that CDH1 expression is heterogeneous in different samples.

We calculated the DMPs of the promoter regions of these four hub genes as shown in Supplementary Table S1. There are CGIs in the promoter region of the CDH1 gene, and several methylation sites were downregulated. Furthermore, we analyzed the correlation between the expression of these four hub genes and methylation sites, as shown in Fig. 2B–N, which shows that the methylation level of the CDC20 promoter region is generally higher, and the methylation levels of the promoter regions are significantly negatively correlated with the expression of these hub genes.

### The relationship between hub genes and survival in EC

**Figure 3:**
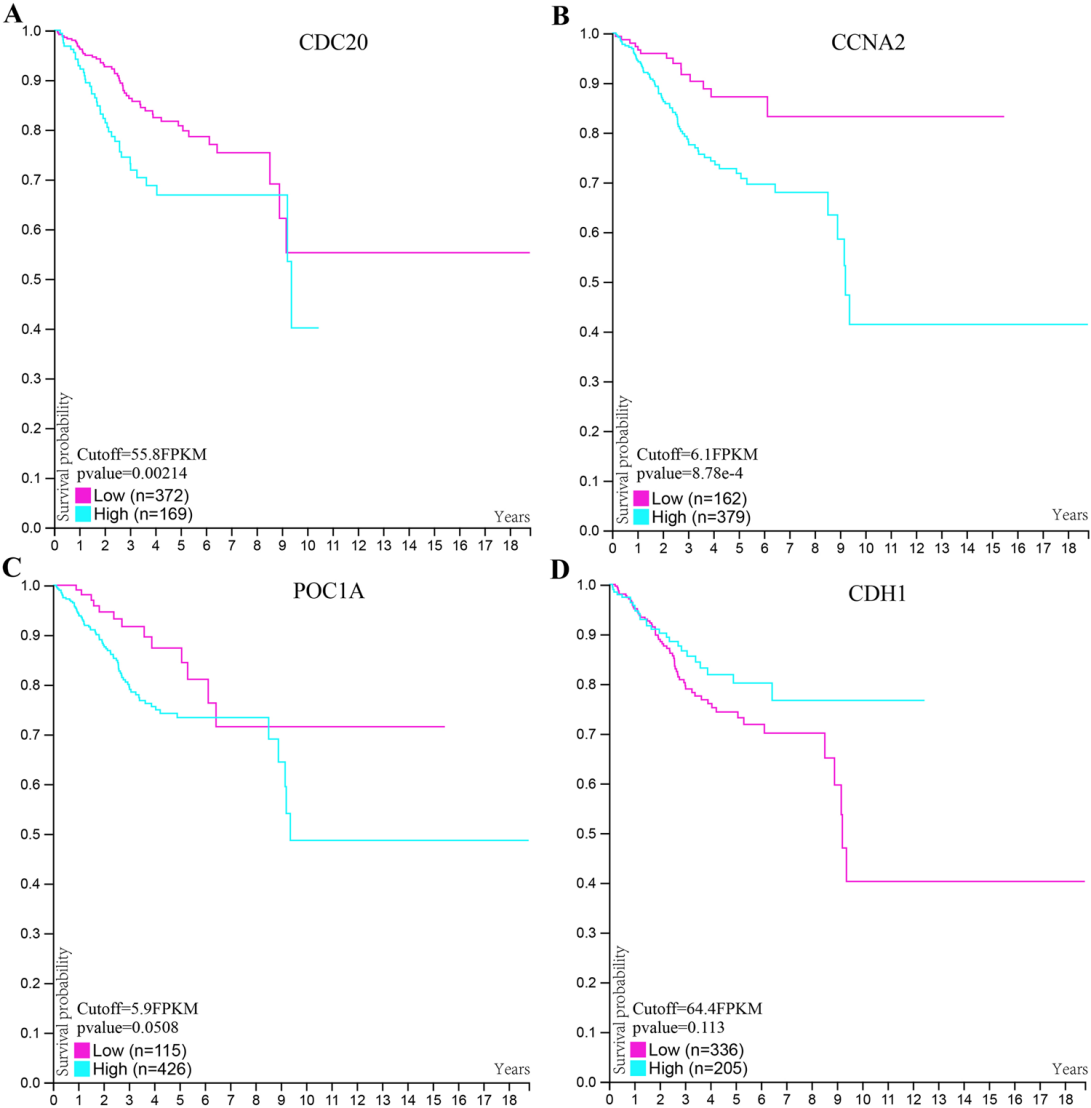
Prognostic analysis of the four hub genes.

**Figure 4:**
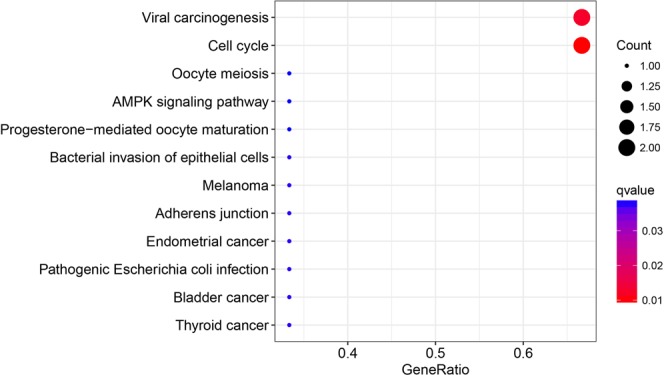
KEGG enrichment analysis of four hub genes.

The prognostic value of the four hub genes was assessed by the Human Protein Atlas (https://www.proteinatlas.org). The threshold was adjusted to a Cox P value < 0.05. Patients with low expression of CDC20 and CCNA2 showed better overall survival in EC (Fig. 3). The GO analysis for these four hub genes revealed that CDC20 and CCNA2 were mainly enriched for the regulation of cell cycle processes, which also showed that CDC20 and CCNA2 played an oncogenic role (Fig. 4).

### Evaluation of the hub genes by IHC

**Figure 5:**
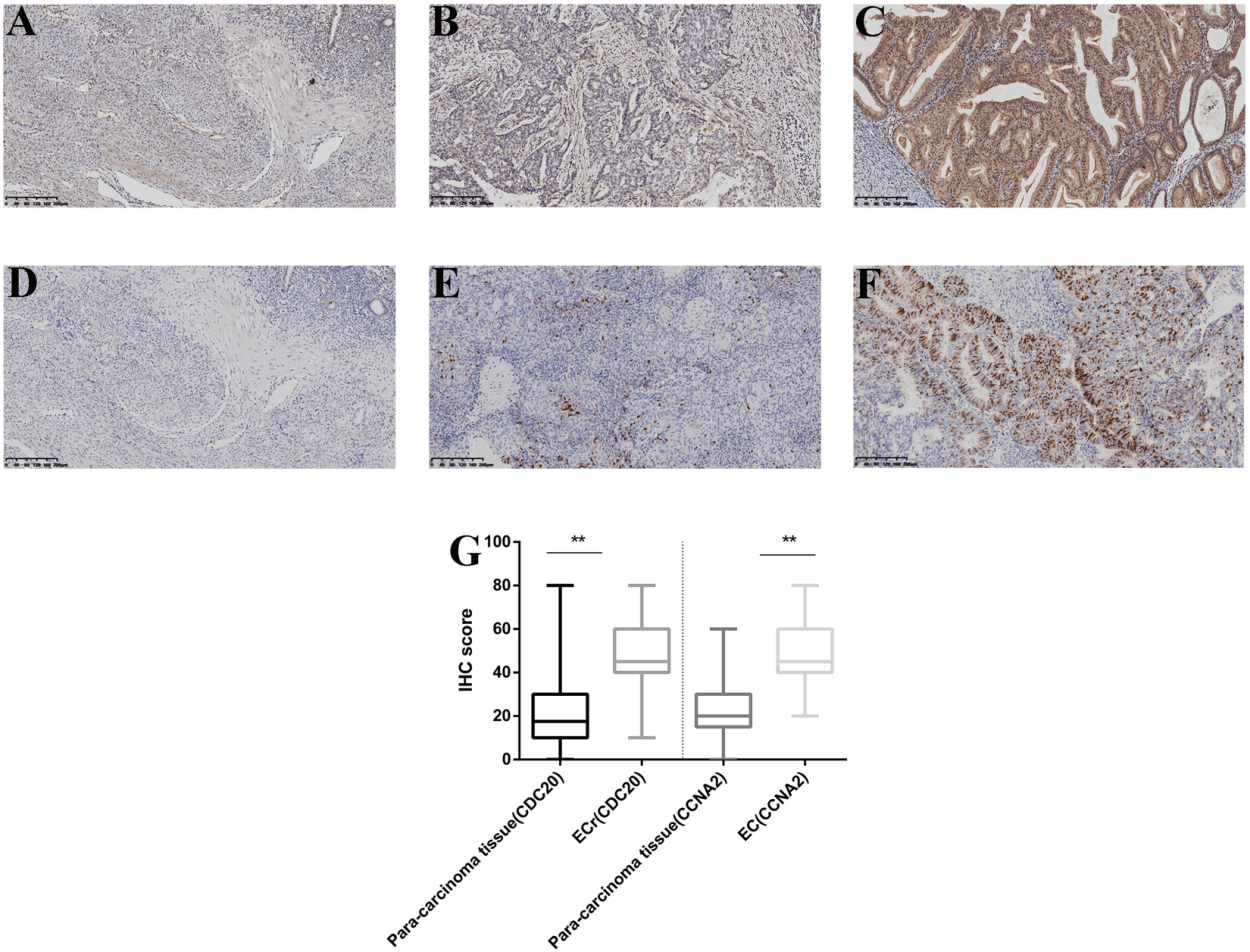
Immunohistochemistry for CDC20 and CCNA2. Samples from endometrial tissue (N = 30) and endometrial cancer (N = 100). Cancer-adjacent endometrial tissue sample of weak immunostaining score for either (**A**) CDC20, (**D**) CCNA2. Endometrial cancer sample of weak and strong immunostaining score for either CDC20 (**B**,**C**), CCNA2 (**E**,**F**). The expression for CDC20 and CCNA2 genes were depicted in (**G**) slides. (X 100).

**Figure 6:**
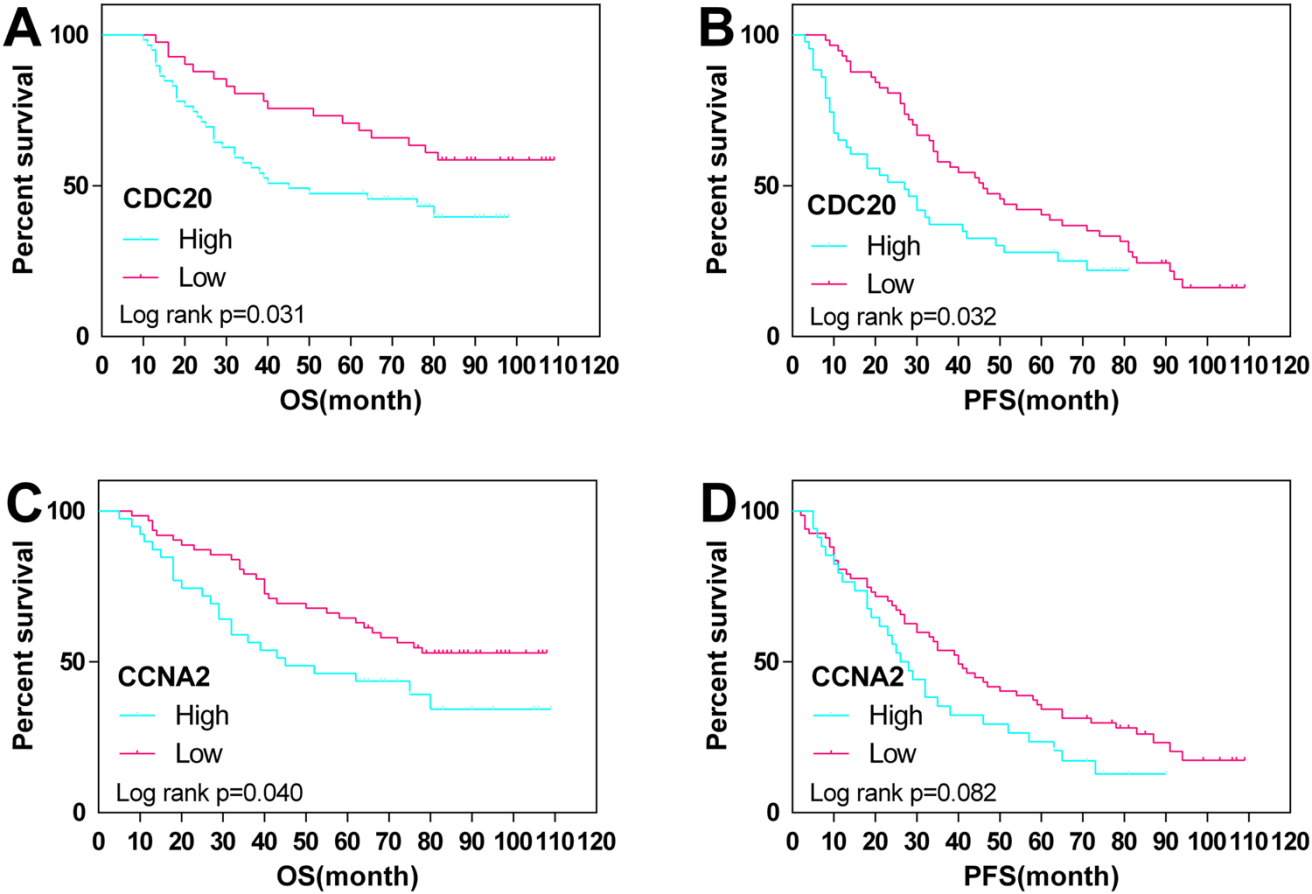
Overall (OS) and disease-free (DFS) survival curves in endometrial cancer (N = 100) according to CDC20 (**A**,**B**) and CCNA2 (**C**,**D**) genes expression status.

From January, 2010 to January, 2013, a total of 130 human endometrial tissue samples, 100 stage III-IV cancer of which had accompanying follow-up information, and 30 cancer-adjacent endometrial tissue samples from archives of paraffin-embedded tissues was collected at the Department of Pathology of Peking Union Medical College Hospital. The follow-up was performed until December 30, 2018. Supplementary Table S2 summarizes the characteristic of all patients, including, age, disease stage, and tumor grade. We selected the two hub genes (CDC20 and CCNA2) that rarely been studied in endometrial cancer to evaluate gene expression values using IHC. The expression differences of CDC20 and CCNA2 between endometrial cancer tissues and adjacent normal endometrial tissues were explored, as shown in Fig. 5. The CDC20 (50.25 ± 1.74 vs 23.67 ± 3.43, p < 0.01) and CCNA2 (46.65 ± 1.44 vs 24.67 ± 2.43, p < 0.01) shows significantly higher expression in endometrial cancer than cancer adjacent tissue. In addition, the correlation between the expression of these genes and the prognosis of endometrial cancer is shown in Fig. 6. These data show that the higher expression of CDC20 (OS, HR = 1.863, 95% CI 1.065–3.195, p = 0.031; PFS, HR = 1.598, 95% CI 1.063–2.759, p = 0.032) and CCNA2 (OS, HR = 1.740, 95% CI 1.034–3.273, p = 0.040; PFS, HR = 1.480, 95% CI 0.955–2.536, p = 0.082) were associated with poor prognosis in endometrial cancer patients.

### Evaluation of the hub genes function

**Figure 7:**
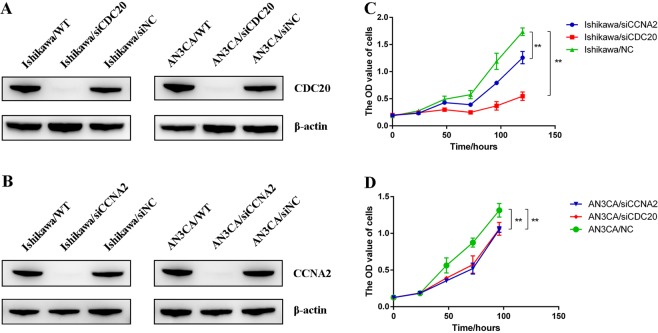
CDC20 and CCNA2 knockdown decrease the cell proliferation in Ishikawa and AN3CA cells. (**A**) CDC20 inhibition via transfection of siCDC20 silenced its protein expression in Ishikawa and AN3CA cells, respectively. (**B**) CCNA2 inhibition via transfection of siCCNA2 silenced its protein expression in Ishikawa and AN3CA cells, respectively. (**C**,**D**) Cell growth curves of Ishikawa and AN3CA. The data are shown as mean ± SD. Statistical significance was determined by *t* test. *P < 0.05; **P < 0.01. Uncropped blots are shown in the Supplementary Fig. S6.

The expression of CDC20 and CCNA2 was significantly down-regulated by transfecting with siCDC20 and siCCNA2 (Fig. 7A) in Ishikawa and AN3CA cells. Results showed that knockdown of CDC20 reduced the proliferation of Ishikawa at the 48 hours (p = 0.03), 72 hours (p < 0.001), 96 hours (p < 0.001), 120 hours (p < 0.001) and AN3CA at the 48 hours (p = 0.006), 72 hours (p = 0.001), 96 hours (p = 0.014) after cell plating, and knockdown of CCNA2 reduced the proliferation of Ishikawa at the 72 hours (p < 0.001), 96 hours (p < 0.001), 120 hours (p < 0.001) and AN3CA at the 48 hours (p = 0.002), 72 hours (p < 0.001), 96 hours (p = 0.006) after cell plating (Fig. 7B).

## Discussion

Despite repeated attempts to better study the molecular mechanisms of EC, the clinical outcome for patients with advanced stage EC remains unsatisfactory, with a 5-year overall survival rate of only 16% to 69%^1^. At present, the combination of surgical-pathological staging according to the FIGO system and histological staging is commonly used to predict the survival of patients. However, the prognosis remains considerably variable for patients at the same stage and having similar pathological features due to the heterogeneity of EC in terms of its clinical behavior and molecular characteristics^2,3^. Although several previous studies have identified more molecular biomarkers for the diagnosis or prognosis of EC, most work has focused on gene expression profiles and protein assays^21,22^. Recently, the detection of DNA methylation has been focused on the diagnosis and progression of EC. Pabalan N *et al*. reported that RASSF1A had good marker potential for EC, and aberrantly methylated regions of RASSF1A demonstrated sensitivity and specificity for the detection of EC in a systematic review and meta-analysis^23^. Chuandi Men *et al*. analyzed DNA methylation and gene expression data from the TCGA database and identified that protocadherin (PCDH) clusters, DDP6, TNXB, and ZNF154, may be novel deregulated genes with altered methylation in EC^24^. However, according to comprehensive research on the diagnostic role of DNA methylation in EC, more specific methylated genes need to be investigated to enhance the diagnostic specificity in clinical samples^25^. Overall, few studies have evaluated the potential predictive role of DNA methylation patterns in EC prognosis. Although TBX2, CHST11, and NID2 were identified as having specific DNA methylation signatures for unfavorable clinical predictive and prognostic factors and a prognostic model that contained 15 methylation markers was recently established to distinguish EC patients with a poorer prognosis^18,19^ biomarkers with higher accuracy should be investigated to assist in prognosis prediction for EC patients.

In the present study, we screened for aberrant DNA methylation and gene expression in EC according to the data from TCGA database. Because hypermethylation often inhibits the expression of downstream genes and hypomethylation often promotes the expression of downstream genes, 329 EI genes and 359 ES genes that could be regulated by aberrant DNA methylation were obtained by the combined analysis of DEGs and DMPs. To further investigate the effect of these EI/ES gene changes in biological processes and pathways, statistically significant GO cluster terms were obtained, and KEGG pathway analysis was performed. Most EI/ES genes are enriched in many core cancers signaling pathways that are known to be important in EC progression, including the cell cycle, the p53 signaling pathway, and proteoglycans in cancer. These results indicated that the aberrant methylation of EI and ES genes is significantly associated with the above core cancer signaling pathways and contributes to carcinogenesis and the progression of EC. Therefore, hub genes were further screened by a genetic interaction subnet, and the prognostic value of the hub genes was further investigated. Most genes in the genetic interaction network tend to be isolated, and only a few hub genes have obvious concentrations of connections with other genes as shown in Supplementary Fig. S4 and S5. A hub gene with higher node degrees is more likely to be a disease-related gene because it could affect downstream biological functions by regulating the expression of adjacent genes in the interaction network^26^. Ultimately, 4 hub genes (CCNA2, CDC20, POC1A and CDH1) were identified, and 2 (CDC20 and CCNA2) were significantly associated with the prognosis of EC. CDC20 is a regulatory protein that appears to interact with several other proteins at multiple points in the cell cycle^27^ which agrees with our GO analysis. Enhanced expression of CDC20 is more often found in various tumor types (including lung adenocarcinoma, breast cancer, bladder cancer and prostate cancer) and might serve as a novel cluster of prognostic biomarkers^28–31^. In addition, studies that focused on abnormal DNA methylation in hepatocellular carcinoma have also shown that patients with hypomethylation and high expression of CDC20 had shorter overall survival and that this gene served as a novel biomarker for precision diagnosis and treatment^32^. Nevertheless, few studies have reported the role of CDC20 in EC, especially about aberrant DNA methylation. CCNA2 belongs to the highly conserved cyclin family, the function of whose members controls both the G1/S and G2/M transition phases of the cell cycle. Over-expressed CCNA2 has been identified in several malignant tissues, such as oral, bladder, and hepatocellular cancer, and may be used as a diagnostic and prognostic biomarker as well as a molecular target for treatment^33–35^. Moreover, Previous study also showed that the high expression of cyclin A, as a cell-cycle regulator, could be a useful marker for poor prognosis of endometrial cancer^36^. In present study, the results also demonstrated that the expression of CDC20 and CCNA2 is significantly associate with cell proliferation. Recently, Zhang *et al*. evaluated prognostic factors based on 328 patients with EC and demonstrated that CCNA2 plays a vital role in EC proliferation and prognosis^37^. However, the aberrant DNA methylation of CCNA2 has rarely been investigated in EC.

## Conclusion

In conclusion, our study integrated the DNA methylation and RNA-Seq data between EC and normal control samples from the TCGA database and identified 2 hub genes involved in core cancer signaling pathways known to be important in EC tumorigenesis. In addition, we also validated the DMPs of CDC20 and CCNA2 using the external TCGA database and demonstrated the utility of aberrant DNA methylation of CDC20 and CCNA2 in predicting the prognosis of EC patients. Moreover, further validation and molecular mechanism studies will be performed at our center.

## Materials and Methods

### Data sources and preprocessing of DNA methylation data

DNA methylation data from 478 samples (431 primary tissue samples, 1 recurrent sample, and 46 paracancerous tissue samples, which contained 33 pairs of matched cancer and adjacent cancer samples) generated using the Illumina Human Methylation 450k Array were obtained from UCSC Xena (https://genome-cancer.ucsc.edu/). RNA-Seq gene expression data from 579 EC samples (543 primary tissue samples, 1 recurrent tissue sample, and 35 paracancerous tissue samples, which contained 23 pairs of matched cancer and adjacent cancer samples.) were obtained from The Cancer Genome Atlas (TCGA, https://cancergenome.nih.gov/). Twenty-one pairs of cancerous and paracancerous samples, which were simultaneously performed for methylation and RNA-seq, were selected to screen for differential methylation and differentially expressed genes. obtained by matching methylation profiles and RNA-Seq data of c samples.

The DNA methylation data were preprocessed with MiNiFi software and normalized with SWAN^38,39^ and then positions that have been shown to be cross-reactive, or demonstrated to map to multiple places in the genome, were filtered out. This list was originally published by Chen *et al*.^40^. CpG sites where the N-A value exceeds 70% were removed from each sample, and the impute.knn of the R package was used to fill in the missing value. Subsequently, unstable genomic methylation sites, including CpG sites and the single nucleotide sites on the sex chromosomes, were removed, and the methylation positions were simultaneously mapped to the gene promoter region, which was defined as 1500 bp upstream to 500 bp downstream of the gene transcription start site (TSS)^38^. Methylation sites that were not labelled as part of the promoter region of genes were removed, and finally, 208,022 CpG sites were left. RNA-Seq data in the Fragments Per Kilobase of exon model per Million mapped fragments (FPKM) format were converted to the Transcripts Per Kilobase of exon model per Million mapped reads (TPM) format.

### Integrated analysis of differentially expressed genes (DEGs) and differentially methylated promoter positions (DMPs)

We calculated the ratio of the median expression of each gene in the matched samples as the fold change and finally selected the DEGs with significant p < 0.05 and |log2(fold change)| > 1 using a paired t test. The DMPs with p < 0.05 were selected. We analyzed the relationship between DEGs and DMPs and defined hypomethylated highly expressed genes as epigenetically induced (EI) genes and hypermethylated lowly expressed genes as epigenetically suppressed (ES) genes in order to screen for significantly negatively related genes as the final EI and ES genes.

### Functional and pathway enrichment analyses for EI and ES genes

Gene ontology (GO) analyses and Kyoto Encyclopedia of Genes and Genomes (KEGG) pathway enrichment analyses were performed for EI and ES genes using the ClusterProfiler package for R to identify over-represented GO terms and statistically significantly enriched pathways.

### Generation and analysis of a genetic interaction subnet

To systematically identify hub genes that are significantly associated with EC, we downloaded all protein interaction data from the Human Integrated Protein–Protein Interaction reference (HIPPIE) v2.0 (https://www.ncbi.nlm.nih.gov/pmc/articles/PMC5210659/) to construct a human protein-protein interaction (PPI) network and subsequently constructed an EI/ES gene interaction subnet by mapping the EI and ES genes into the PPI network.

Biological networks are complex networks often characterized by having self-organization, self-similarity, attractor, small world theory and scale-free features. We used the PPI network as a background to analyze the degree distribution of EI/ES gene interaction subnets and evaluate whether the EI/ES gene interaction subnet conformed to the scale-free feature. We calculated the number of genes connected to each EI/ES gene, the number of genes interacting with each other and the number of connected genes using the PPI as a statistical background to construct a statistical model that could perform Fisher’s enrichment test for each gene and screen the significant network nodes with a false discovery rate (FDR) < 0.01 and number of interaction nodes > 5 as the hub EI/ES gene.

### The validation of differentially methylated CpG sites (DMCs) and differentially methylated regions (DMRs) and Kaplan–Meier analysis of hub genes

The DMCs and DMRs of the hub EI/ES genes between EC and adjacent normal tissues were also analyzed by an external 27 K dataset, which was downloaded from TCGA and contained 10 paired cancer and normal samples for gene expression profiling and 12 paired cancer and normal samples for methylation profiling. To further explore the relationship between the expression of the hub genes and prognosis of EC, TCGA RNA-Seq data and survival analysis were performed by the Human Protein Atlas (https://www.proteinatlas.org) online tool and the Kaplan–Meier curve, respectively.

### Immunohistochemical staining (IHC)

We collected a total of 130 human endometrial tissue samples, 100 stage III-IV cancer tissue of which had accompanying follow-up information, and 30 cancer-adjacent endometrial tissue samples from archives of paraffin-embedded tissues between January, 2010 and January, 2014 at the Department of Pathology of Peking Union Medical College Hospital. The follow-up was performed until December 30, 2018. The pathological diagnoses were reconfirmed by a pathologist. The project was approved by the Ethical Committee (Peking Union Medical College Hospital), and informed consent was acquired from patients or family members. IHC was performed as previously described^41^. Anti-antibody (CDC20 1:100, Abcam, ab86104, CCNA2 1:400, Abcam, ab181591) was used for IHC. The scoring details have been described previously^42^.

### Cell culture and cell proliferation detection

Human endometrial cancer cell lines (Ishikawa and AN3CA) were purchased from Institute of Basic Medical Sciences, Chinese Academy of Medical Sciences (Bei Jing, China). Ishikawa, AN3CA were cultured in DMEM (10% fetal bovine serum) at 37 °C, 5% CO2 condition. siCDC20 and siCCNA2 purchased from Guangzhou Ribobio Co., Ltd. and transfected into cells by Lipofectamine RNAiMAX. The target sequence of siCDC20 was 5′-GACCACTCCTAGCAAACCT-3′, the target sequence of siCCNA2 was 5′-GCTGTGAACTACATTGATA-3′. After 48 hours of transfection, western blot was used to detect the efficiency of gene knockdown. The western blot was performed as previously described^43^. The primary antibodies were anti-CDC20 (1:1000, Abcam, ab26483), anti-CCNA2 (1:1000, Abcam, ab181591) and anti-β-actin (1:1000, Abcam, USA). After 48 hours of transfection, cell growth curve (Cell Counting Kit-8 assay) was used to evaluate the proliferation of each group of cells. The CCK-8 assay was performed as previously described^44^.

## Supplementary information

Supplementary Information
