# Supplementary material for: Identification of prognosis markers for endometrial cancer by integrated analysis of DNA methylation and RNA-Seq data
Source: Sci Rep. 2019 Jul 9;9:9924. doi: 10.1038/s41598-019-46195-8 (PMC6617448; doi:10.1038/s41598-019-46195-8)
Supplement: Supplementary file 1 — Supplementary Information [file 41598_2019_46195_MOESM1_ESM.docx]

**Identification of prognosis markers for endometrial cancer by integrated analysis of DNA methylation and RNA-Seq data**

Xiao Huo^1a^, Hengzi Sun^1a^, Dongyan Cao^1^, Jiaxin Yang^1^, Peng Peng^1^, Mei Yu^1^, Keng Shen^1 *^

Author affiliations

^1^ Author affiliations: Department of Obstetrics and Gynecology, Peking Union Medical College Hospital, Chinese Academy of Medical Sciences and Peking Union Medical College, Beijing, China;

^a^ The first two authors contributed equally.

Correspondence: Keng Shen

Department of Obstetrics and Gynecology, Peking Union Medical College Hospital, No. 1 ShuaiFuYuan, Dongcheng District, Beijing, China 100730

Tel: 86-10-69155200

Fax: 86-10-69154022

Correspondence email: shenkengpumc@163.com

**Supplemental information**

**Supplementary Figure S1.** The distribution of genes corresponding to different differentially promoter methylation positions numbers and identification of aberrantly methylated and differentially expressed genes. (A) The distribution of all genes; (B) Hypomethylated highly expressed and hypermethylated lowly expressed genes; (C) The distribution of negatively regulated genes.


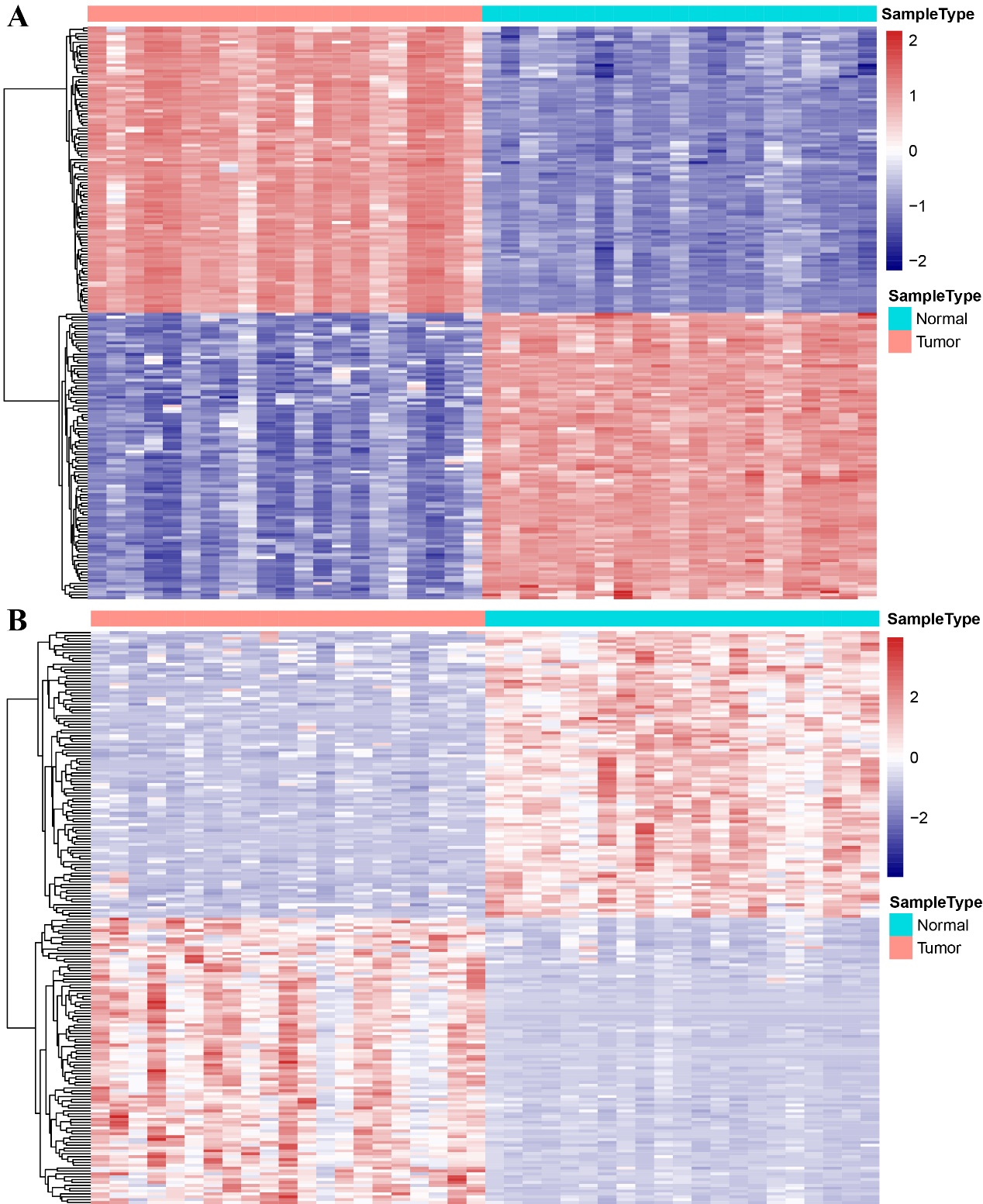


**Supplementary Figure S2.** The KEGG enrichment analysis of epigenetically induced and epigenetically suppressed genes. (A) Epigenetically induced genes; (B) Epigenetically suppressed genes.


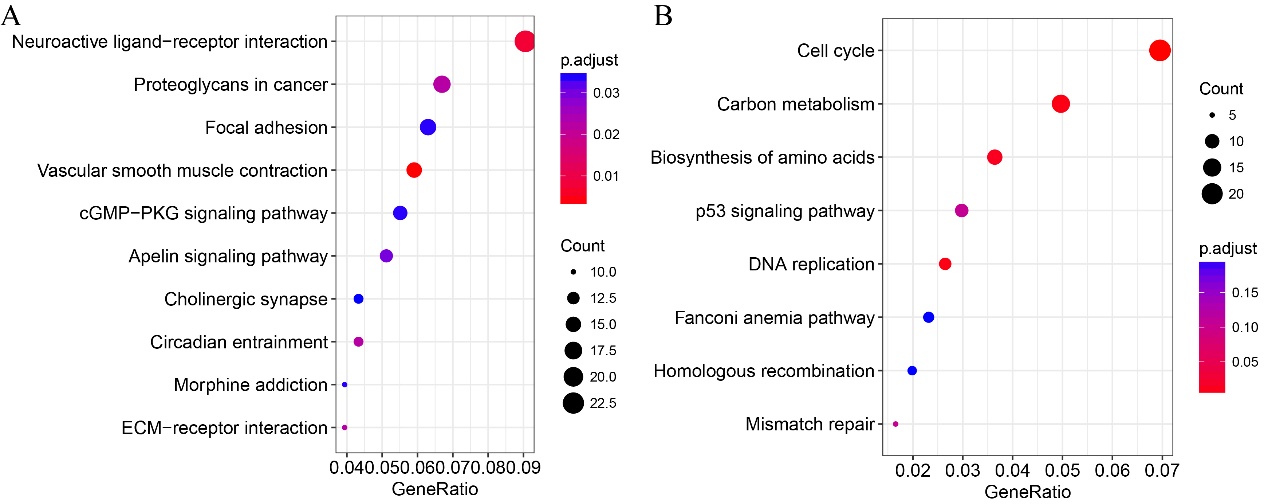


**Supplementary Figure S3.** The GO analysis of epigenetically induced and epigenetically suppressed genes. (A) Epigenetically induced genes; (B) Epigenetically suppressed genes.


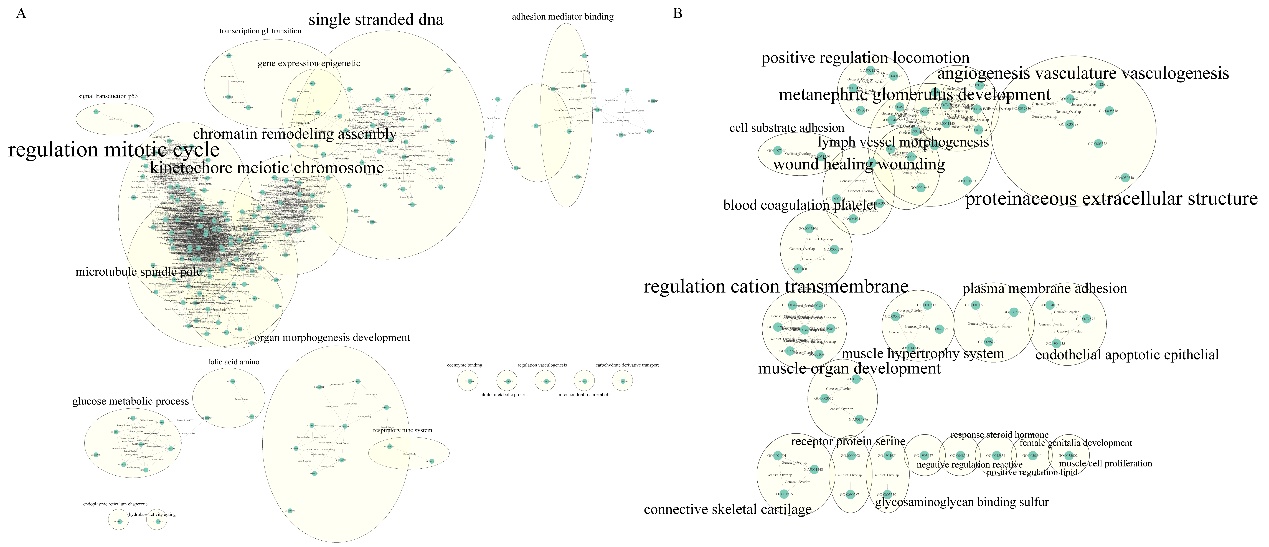


**Supplementary Figure S4.** Degree distribution of the network. (A) The degree distribution of the background network; (B): The degree distribution of the epigenetically induced and epigenetically suppressed genes interaction network.


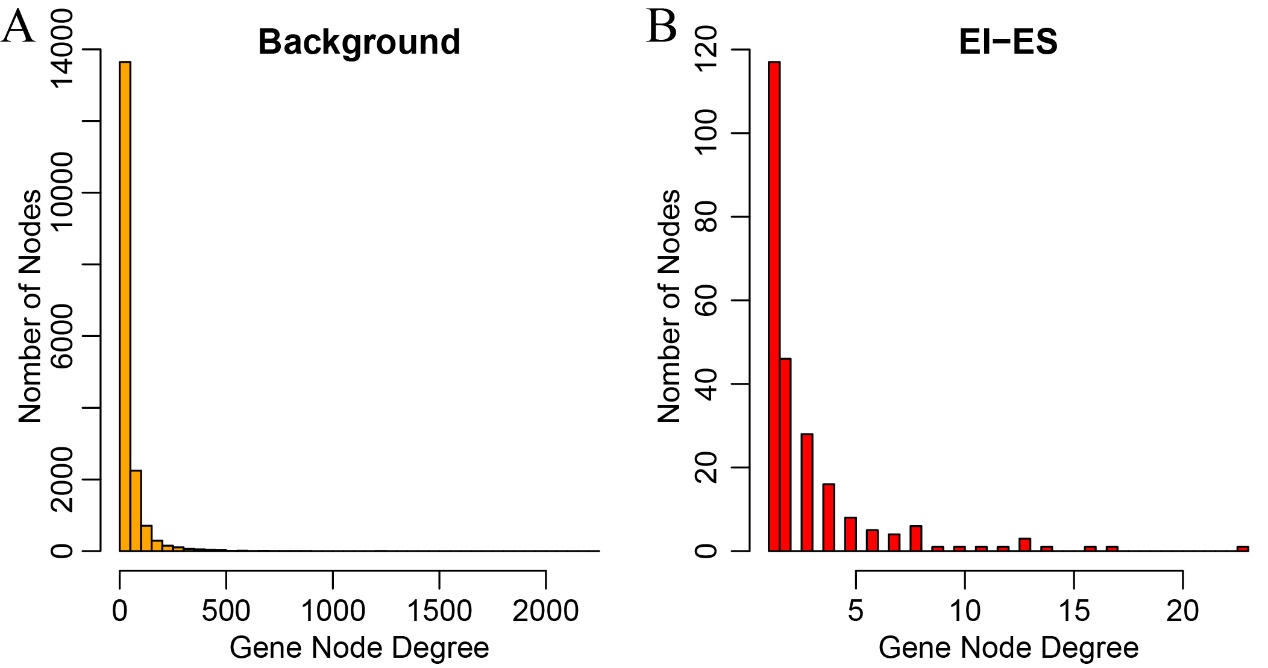


**Supplementary Figure S5.** The genetic interaction subnet of epigenetically induced and epigenetically suppressed genes


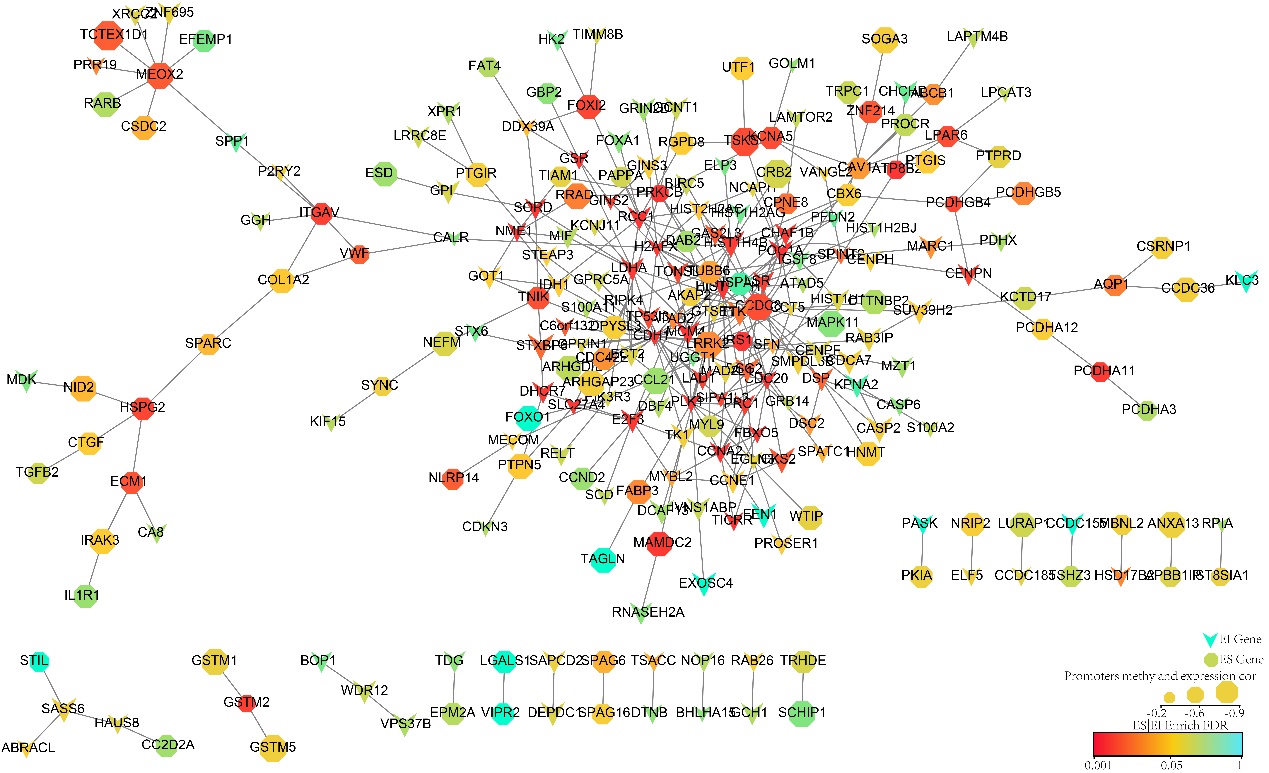


**Supplementary Figure S6.** Full length blots of CDC20 in Ishikawa cells(A) and AN3CA(B) after knockdown experiments, and corresponding β-actin(C, D). Full length blots of CCNA2 in Ishikawa cells(E) and AN3CA(F) after knockdown experiments, and corresponding β-actin(G, H).
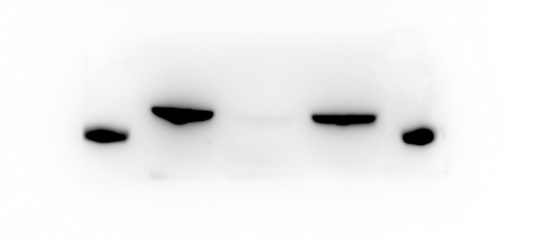

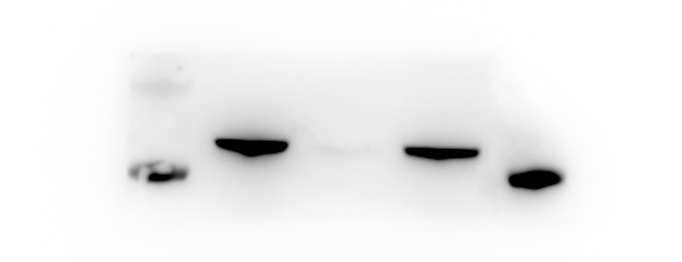


**A**

**B**


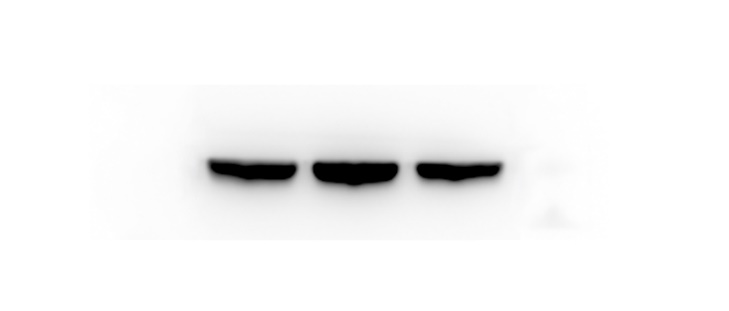


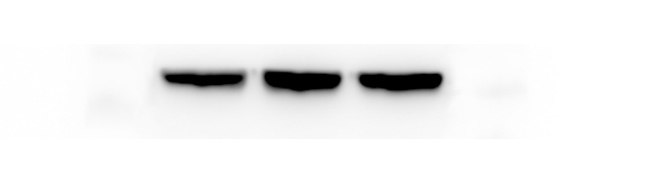


**D**

**C**

**
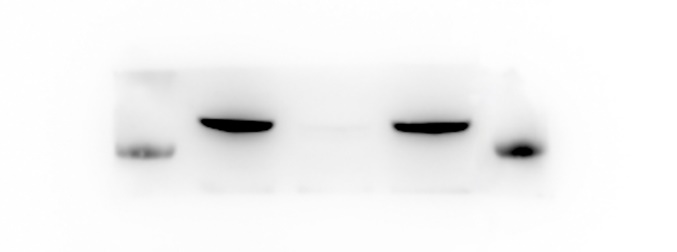

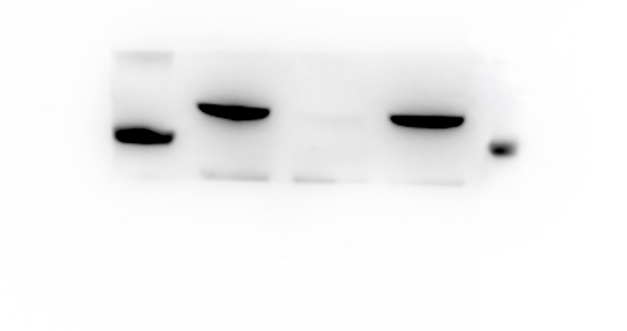
**

**F**

**E**

**H**

**G**

**
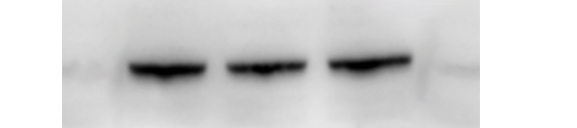

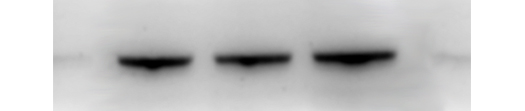
**

**Supplementary Table S1.** Differential methylation positions of the four hub genes.

| **CpG** | **Chrom** | **Start** | **End** | **GeneSymbol** | **Feature_Type** |
| --- | --- | --- | --- | --- | --- |
| cg00238349 | chr3 | 52153775 | 52153776 | POC1A | N_Shore |
| cg15003208 | chr3 | 52155201 | 52155202 | POC1A | S_Shore |
| cg26316544 | chr3 | 52154402 | 52154403 | POC1A | Island |
| cg12718671 | chr4 | 1.22E+08 | 1.22E+08 | CCNA2 | N_Shore |
| cg05525368 | chr1 | 43360233 | 43360234 | CDC20 | S_Shore |
| cg06373377 | chr1 | 43360074 | 43360075 | CDC20 | S_Shore |
| cg26607641 | chr1 | 43358985 | 43358986 | CDC20 | Island |
| cg04398983 | chr16 | 68737189 | 68737190 | CDH1 | Island |
| cg05785947 | chr16 | 68737156 | 68737157 | CDH1 | Island |
| cg11255163 | chr16 | 68737297 | 68737298 | CDH1 | Island |
| cg11667754 | chr16 | 68737078 | 68737079 | CDH1 | N_Shore |
| cg17655614 | chr16 | 68737041 | 68737042 | CDH1 | N_Shore |
| cg23989635 | chr16 | 68737300 | 68737301 | CDH1 | Island |

**Supplementary Table S2.** Characteristics of endometrial cancer patients.

| **Characteristic** | **No.** | **%** |
| --- | --- | --- |
| **Age (y), median (range)** | 55.5(36-74) | 100% |
| **Stage** |  |  |
| III | 74 | 74.0% |
| IV | 26 | 26.0% |
| **Histological subtype** |  |  |
| Endometroid | 100 | 100% |
| **Differentiation grade** |  |  |
| G1 | 12 | 12.0% |
| G2 | 45 | 45.0% |
| G3 | 28 | 28.0% |
| Unknown | 15 | 15.0% |
| **Residual disease** |  |  |
| optimal | 85 | 85.0% |
| suboptimal | 10 | 10.0% |
| unknown | 5 | 5.0% |
